# A Sensitivity Enhanced MWCNT/PDMS Tactile Sensor Using Micropillars and Low Energy Ar^+^ Ion Beam Treatment

**DOI:** 10.3390/s16010093

**Published:** 2016-01-12

**Authors:** Syed Azkar Ul Hasan, Youngdo Jung, Seonggi Kim, Cho-Long Jung, Sunjong Oh, Junhee Kim, Hyuneui Lim

**Affiliations:** 1 Department of Nature-Inspired Nano Convergence Systems, Korea Institute of Machinery and Materials, Daejeon 34103, Korea; azkarulhasan@kimm.re.kr (S.A.U.H.); yjung@kimm.re.kr (Y.J.); choucreme85@kimm.re.kr (S.K.); andend6@kimm.re.kr (C.-L. J.); ssun@kimm.re.kr (S.O.); junhee_kim@kimm.re.kr (J.K.); 2 Department of Nanobiotechnology, University of Science & Technology, Daejeon 34113, Korea

**Keywords:** MWCNT/PDMS, piezoresistive tactile sensor, micropillar, ion beam treatment, curved pillar, directional detection

## Abstract

High sensitive flexible and wearable devices which can detect delicate touches have attracted considerable attentions from researchers for various promising applications. This research was aimed at enhancing the sensitivity of a MWCNT/PDMS piezoresistive tactile sensor through modification of its surface texture in the form of micropillars on MWCNT/PDMS film and subsequent low energy Ar^+^ ion beam treatment of the micropillars. The introduction of straight micropillars on the MWCNT/PDMS surface increased the sensitivity under gentle touch. Low energy ion beam treatment was performed to induce a stiff layer on the exposed surface of the micropillar structured MWCNT/PDMS film. The low energy ion bombardment stabilized the electrical properties of the MWCNT/PDMS surface and tuned the curvature of micropillars according to the treatment conditions. The straight micropillars which were treated by Ar^+^ ion with an incident angle of 0° demonstrated the enhanced sensitivity under normal pressure and the curved micropillars which were treated with Ar^+^ ion with an incident angle of 60° differentiated the direction of an applied shear pressure. The ion beam treatment on micropillar structured MWCNT/PDMS tactile sensors can thus be applied to reliable sensing under gentle touch with directional discrimination.

## 1. Introduction

Human skin is the large, flexible, stretchable and sensitive organ [1,2]. The realization of flexible tactile sensing devices mimicking the human skin has elicited tremendous research interest for various applications such as bionic arms or cellphone touch panels. Nanocomposites have demonstrated their potential for incorporating a tactile sensing functionality similar to the mechanoreceptors present beneath the human skin [3]. Based on the transduction mechanism, tactile sensing units can be broadly categorized as capacitive [4,5,6,7,8,9,10,11], piezoelectric [12,13,14,15,16,17], piezoresistive [18,19,20,21,22,23,24] and triboelectric types [25,26], and each transduction type has its own advantages and disadvantages. Among them, piezoresistive tactile sensing devices show the advantages of flexibility, mechanical robustness, insensitivity to overload, and less complicated electronics requirements [27]. Especially, the nanocomposites having both the high conductivity associated with nanoscale conductive fillers in the form of carbon black, graphite and multi-walled carbon nanotubes (MWCNTs), and the high elasticity possessed by polymer elastomers such as polydimethylsiloxane (PDMS) are good candidates for exhibiting piezoresistive behavior for tactile sensing in electronic skins [28,29].

Tuning and enhancing the sensitivity for tactile sensing have been conceived as major research landmarks towards optimized tactile sensor performance. Lai *et al.* [30] have achieved sensitivity with tunable ranges by varying the driving frequency of sensing arrays comprised of a sensing material in the form of carbon nanotubes (CNTs), dispersed inside nematic liquid crystals, deformable PDMS structures, indium tin oxide (ITO) glass substrates and ITO-coated polyethylene terephthalate (PET) films. Pan *et al.* [31] successfully enhanced the sensitivity of a resistive pressure sensor through the use of interconnected hollow sphere microstructures inside a rigid conducting polymer. The role of microstructures on surface in the form of microdomes on the MWCNT/PDMS film for demonstrating higher sensitivity was highlighted by Park *et al.* [32]. They also utilized the interlocked microdomes of two MWCNT/PDMS films facing each other for higher sensitivity compared to that achievable with a single film. In a subsequent study, the utility of a surface texture in the form of micropillars over the MWCNT/PDMS film was demonstrated. It was revealed that micropillar structured MWCNT/PDMS with shorter pitch was more sensitive as compared to that having longer pitch, but the height of micropillars was limited to 6 μm [33]. The sensitivity of nanocomposite films can be affected by the morphology of the microstructures on the surface such as height, pitch, diameter, shape, *etc.* There are several studies aiming to modulate micropillar morphology. Especially, simple ion beam treatment can change the micropillar shape, which can then show different responses to directional touch. The microstructures in the form of curved PDMS micropillars were demonstrated by using Ar^+^ ion beam irradiation in order to mimic the anisotropic adhesion pertaining to the feet of gecko [34].

In this paper, we have developed a MWCNT/PDMS piezoresistive tactile sensor skin having surface texture in the form of micropillars as high as 20 μm and showed that the increase in the height of micropillars is another aspect for improving the sensitivity in MWCNT/PDMS piezoresistive tactile sensors. Low energy Ar^+^ ion beam treatment was carried out to generate stiff straight and curved-shape micropillars. The effects of ion beam treatment were investigated via resistance measurements and scanning electron microscopy observations in terms of the electrical properties of the MWCNT/PDMS film, shape changes of the micropillars, and tactile sensing of micropillar structured MWCNT/PDMS tactile sensor under normal pressure and shear pressure.

## 2. Working Principle

The first proposed approach for enhancing the sensitivity of a MWCNT/PDMS tactile sensor is creation of micropillar surface textures as those surface structures can increase the deformation of the sensing elements *i.e.*, MWCNT/PDMS film when normal pressure is applied on the tactile sensor. The second proposed approach is modification of micropillar structured MWCNT/PDMS tactile sensor with low energy Ar^+^ ion beam irradiation as it can strengthen the mechanical stiffness of micropillars and stabilize electrical resistance of the exposed MWCNT/PDMS films. Furthermore, the ion beam irradiation can also generate a strain mismatch between the ion beam exposed part and unexposed area so as to tune the shape of micropillars. These curved-shape micropillars can show different behaviors under shear pressures of various directions.

**Figure 1:**
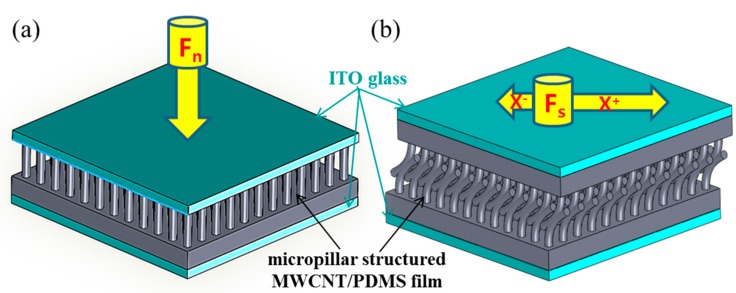
3D schematics of the concept of MWCNT/PDMS tactile sensors; (**a**) enhancing the sensitivity of a single MWCNT/PDMS film-based tactile sensor with stiff micropillar surface texture under applied normal pressure; (**b**) directional sensitivity of a double MWCNT/PDMS film-based tactile sensor having interlocked curved-shape micropillars under applied shear pressure (the longer arrow means a larger resistance change under same magnitude of applied shear pressure).

Figure 1 shows a 3D schematic of the proposed MWCNT/PDMS tactile sensors. The MWCNT/PDMS tactile sensor is composed of MWCNT/PDMS films and two ITO coated glass electrodes. Figure 1a shows the concept of a surface textured MWCNT/PDMS tactile sensor for enhanced sensitivity under applied normal pressure. The micropillar structured MWCNT/PDMS tactile sensor can enhance the sensitivity due to the surface morphology of high aspect ratio micropillars and the thin stiff layers created on the exposed surface of MWCNT/PDMS film after low energy Ar^+^ ion beam treatment. Compared with planar MWCNT/PDMS tactile sensors, micropillar structured MWCNT/PDMS tactile sensors change their resistance more sensitively as the micropillars on the surface deform themselves are more responsive to applied gentle normal pressure. Figure 1b conceptualizes the force direction discrimination possible with a curved micropillar MWCNT/PDMS tactile sensor. The tactile sensor consists of two MWCNT/PDMS films with curved micropillar surface texture, whose micropillars are interlocked each other. When a shear pressure is applied along the interlocking direction, the resistance of the MWCNT/PDMS tactile sensor will decrease due to the tight and strong contact between the interlocked curved micropillars, whereas, the resistance of the MWCNT/PDMS tactile sensor will increase upon application of a shear force against the interlocking direction. The fastening and untangling motion of the interlocking stuctures in the curved micropillars will definitely provide different results in the electrical response of the MWCNT/PDMS tactile sensor under various shear pressures from different directions.

## 3. Fabrication Process

**Figure 2:**
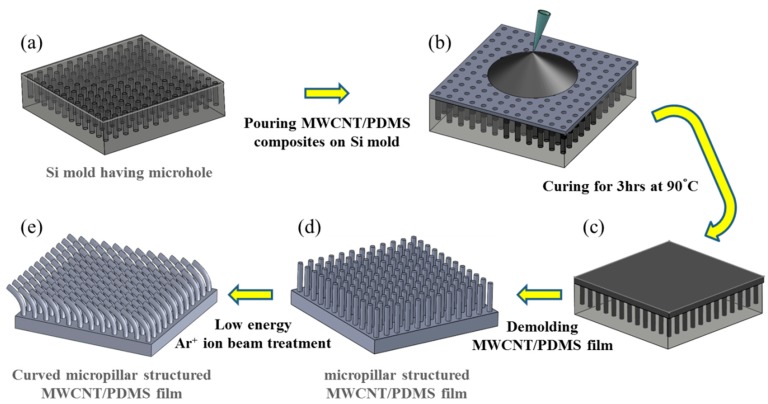
Schematic diagram of the fabrication process for the micropillar structured MWCNT/PDMS film. (**a**) Si mold having microhole arrays prepared by a photolithography and etching process. The depth of microholes corresponds to the height of micropillars; (**b**) pouring MWCNT/PDMS solution onto Si mold; (**c**) curing of MWCNT/PDMS solution on a hot plate at 90 °C for 3 h after removing hexane and entrapped air bubbles; (**d**) demolding of the cured MWCNT/PDMS film with straight micropillar surface texture; (**e**) surface modification of micropillar structured MWCNT/PDMS film using Ar^+^ ion beam irradiation.

**Figure 3:**
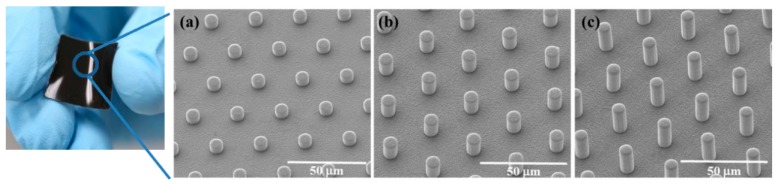
Picture of the fabricated MWCNT/PDMS film (20 mm × 20 mm) and FESEM images of (**a**) 5 μm; (**b**) 10 μm and (**c**) 20 μm high micropillars on MWCNT/PDMS films.

Figure 2 illustrates five steps in the fabrication of MWCNT/PDMS film having straight and curved micropillar surface textures. First, a Si mold with 20 mm × 20 mm dimensions was fabricated through a traditional photolithography and etching process to make microhole arrays of different depths. Then, anti-stiction coating of self-assembly monolayers (SAMs) of trichloro(1*H*,1*H*,2*H*,2*H*-perfluorooctyl)silane (FOTS) was followed. Coating FOTS SAMs on the Si mold provides hydrophobicity on the Si surface and helps the detachment of the composite film even when MWCNT/PDMS film has high aspect ratio micropillars. An 8 wt.% MWCNT/PDMS composite prepolymer was prepared with MWCNT (Sigma-Aldrich Co. LLC., St. Louis, MO, USA) and PDMS prepolymer (Sylgard^®^ 184, Dow Corning Co., Midland, MI, USA). The composite prepolymer of 650 mg was dissolved in 1 mL of *n*-hexane (Samchun Pure Chemical Co., Ltd., Pyeongtaek-si, Gyeonggi-do, Korea) and mixed using vortex mixing for 3 min. The composite solution was poured onto the surface of the Si mold. The air bubbles and hexane trapped in the microhole arrays and composite prepolymer itself were removed inside a vacuum desiccator for 30 min. Finally, the nanocomposite solution was cured on a hot plate at 90 °C for 3 h and cured MWCNT/PDMS films were demolded from the Si mold with the surface texture in the form of vertical micropillars (diameter: 5 μm, pitch: 20 μm, height: 5, 10, and 20 μm), as shown in Figure 3. The morphology of the micropillar structured MWCNT/PDMS film was observed using a field emission scanning electron microscope (FESEM, Nova NanoSEM 200, FEI Co., Hillsboro, OR, USA).

**Figure 4:**
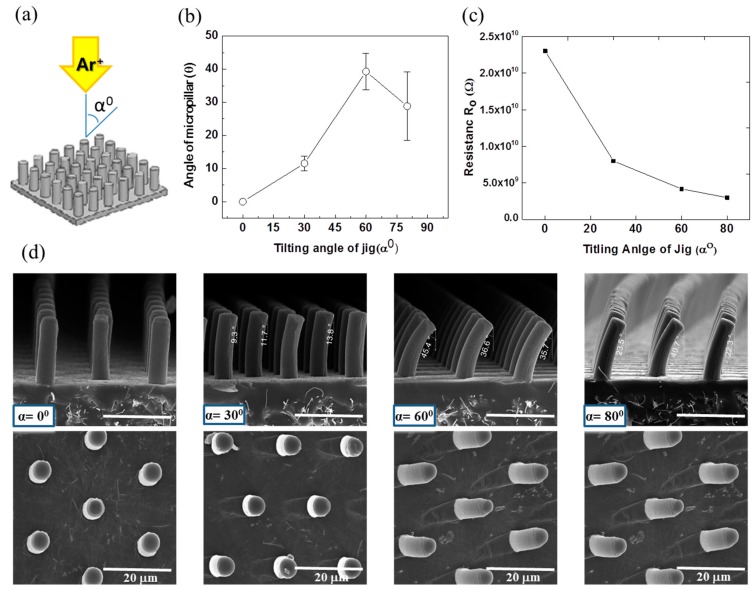
(**a**) Schematic of Ar^+^ ion beam irradiation with different incident angles; (**b**) the angles of curved micropillars according to the incident angles of ion beam irradiation; (**c**) initial resistances of the curved micropillar structured MWCNT/PDMS films according to the incident angles of ion beam irradiation; (**d**) FESEM images of the micropillars according to the incident angles of ion beam irradiation at 0°, 30°, 60° and 80° in side view (upper images) and top view (bottom images).

The developed MWCNT/PDMS film surface having straight micropillars of 20 μm height was modified with low energy Ar^+^ ion beam irradiation in a custom-built sputtering vacuum chamber. The applied voltage, current and exposure time of Ar^+^ ion beam irradiation were 0.1 kV, 0.5 A and 15 min, respectively. The incident angle of the Ar^+^ ion beam irradiation was varied at 0°, 30°, 60° and 80° to tune the curvature of the micropillars, as shown in Figure 4. Ar^+^ ion beam treatment is capable of generating a stiff layer of nanoscale dimensions on the surface of MWCNT/PDMS without affecting the properties of the bulk polymer. The strain mismatch between the thin stiff surface layer and bulk polymer induced the various curvatures of MWCNT/PDMS micropillars as well as wrinkles on the surface of the MWCNT/PDMS film. As the incident angle of the Ar^+^ ion beam irradiation increased, the angle of micropillar curvature bending towards the direction of Ar^+^ ion beam also increased. However, the maximum curvature was achieved in the case of 60° and the curvature of the micropillars decreased when the angle of Ar^+^ ion beam irradiation exceeded 60°. These phenomena may originate from the fact that the sidewalls of the micropillars are completely exposed with an ion beam of 60°, but the shadow effect of neighboring micropillars with oblique ion beam irradiation at incident angles exceeding 60° may prevent the exposure of micropillars to the ion beam.

An increase in the initial resistance, R_o_ of the planar MWCNT/PDMS film was obtained after Ar^+^ ion beam treatment, as shown in Table 1. The initial resistance R_o_ of the planar MWCNT/PDMS film was measured as 1.2 × 10^6^ Ω under a very light pressure of 5 Pa before the ion beam treatment, but the initial resistance R_o_ of the ion beam treated planar MWCNT/PDMS film increased to 2.8 × 10^7^ Ω. This phenomenon is caused by the degradation of C=C bonds, the loss of carbon (C) and hydrogen (H) in MWCNT and the cross-linking of silicon (Si) in PDMS. It was reported that ion beam irradiation on PDMS cannot form a graphite-like carbonized structure due to the presence of high levels of silicon [35]. The same results were also obtained in our experiments through the surface analysis; energy dispersive X-ray spectroscopy (EDS, Octane Pro, EDAX Co., Mahwah, NJ, USA) and X-ray photoelectron spectroscopy (XPS, MultiLab. ESCA 2000, VG Microtech Co., East Grinstead, West Sussex, UK). It was detected that the C/Si ratio decreased after ion beam treatment. In addition, the relative deviation of the initial resistances between the planar PDMS-MWCNT film samples was reduced after ion beam treatment. Moreover, the initial resistance values of micropillar structured MWCNT/PDMS films varied according to the incident angles of the ion beam irradiation, as shown in Figure 4c. As the incident angle of the ion beam was increased, the ion beam penetration depth in the MWCNT/PDMS film decreased such that the amount of carbon degradation and silicon cross-linking decreased. The MWNCT/PDMS film exposed to a low incidene angle ion beam had lower C/Si ratio and higher initial resistance than the film exposed to a high incident angle beam. Therefore, Ar^+^ ion beam irradiation induces both the curvature of the micropillars due to the mismatch of stiffness of the surface and the stable and a relatively high initial resistance due to the change in chemical compositions and bonds.

**Table 1:** Initial resistance (R_o_) of planar PDMS-MWCNT film before and after Ar^+^ ion beam treatment.

Planar PDMS-MWCNT Film	Initial Resistance R_o_ (Ω)
Pristine	1.2 × 10^6^ ± 1.30 × 10^5^
After Ar^+^ ion beam irradiation	2.8 × 10^7^ ± 3.04 × 10^5^

## 4. Results and Discussion

The normal and shear tactile sensing characteristics of the proposed MWCNT/PDMS tactile sensor were analyzed. First, we investigated the sensitivity of ion beam treated MWCNT/PDMS tactile sensors having surface with planar morphology, and micropillars (5, 10, 20 μm high) upon the applied normal pressure. Secondly, the effect on the normal pressure sensitivity according to the curvature of micropillars (20 μm high) induced by ion beam treatment was explored and analyzed to find the MWCNT/PDMS tactile sensor showing the highest sensitivity. Finally the shear sensing capability of detecting the magnitude and direction of the applied shear pressure was studied by applying various types of shear pressures on the MWCNT/PDMS tactile sensors having curved micropillars.

### 4.1. Normal Tactile Sensing Characteristics

#### 4.1.1. Effect of the Micropillar Height 

**Figure 5:**
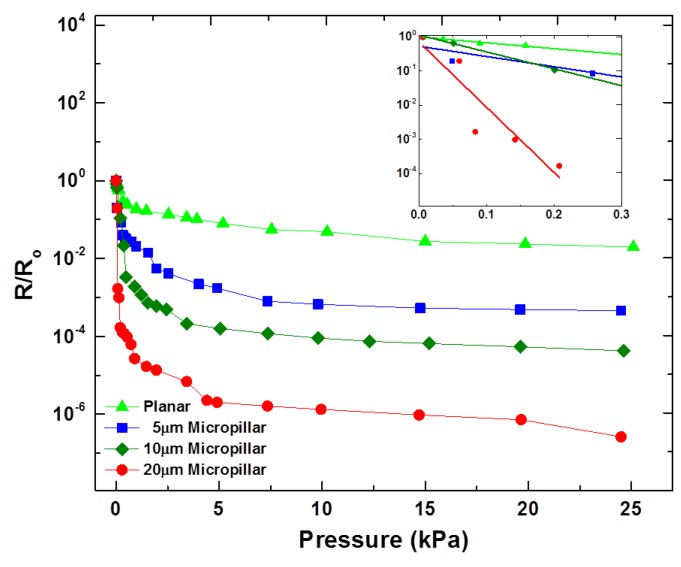
Effect of micropillar height on the sensitivity of the MWCNT/PDMS tactile sensor. The inset shows sensitivities of the developed sensors in the lower pressure regime (<0.3 kPa).

**Figure 6:**
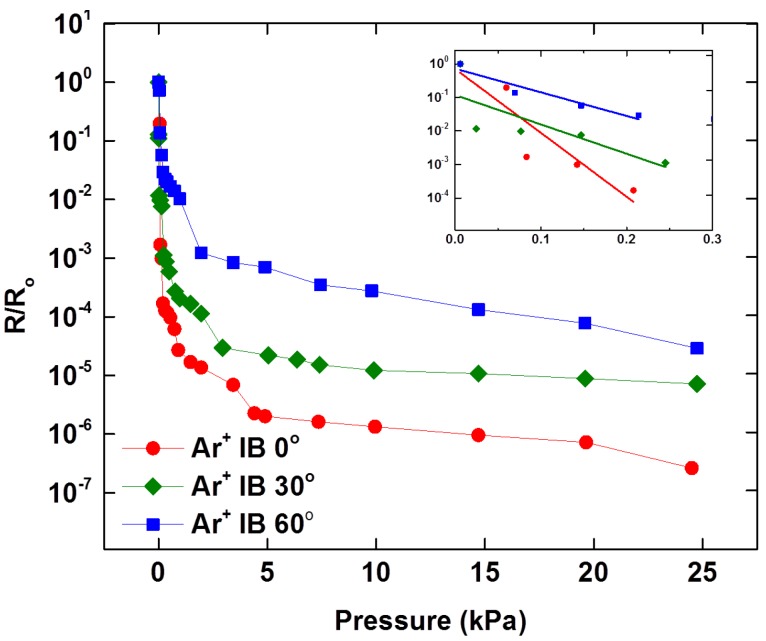
Normal pressure responses of MWCNT/PDMS tactile sensor having 20 μm high micropillars processed by Ar^+^ ion beam treatment at incident angles of 0°, 30°and 60°. The inset shows the sensitivities of the developed sensors in the lower pressure regime (<0.3 kPa).

The normal tactile sensing characteristics of the proposed tactile sensor were determined with a custom-made instrument comprised of a highly sensitive microbalance, a linear motor-based XY stage including a Z-axis manipulator capable of applying normal pressure through the movement of the manipulator tip in the Z-axis direction. The movement in the Z-axis can be controlled with a resolution of 0.1 μm. The resistance changes of the MWCNT/PDMS tactile sensors according to the applied normal pressure were recorded using a source meter (6430, Keithley Instruments Inc., Cleveland, OH, USA). To test the normal tactile sensing capability of the proposed sensor, one electrode was placed on the bottom XY stage of the instrument and the other electrode was attached on the Z-axis manipulator tip. As the Z-axis manipulator moves down, the normal pressure on the tactile sensor increases. The Z-axis manipulator was controlled in step mode and normal pressure of up to 25 kPa was applied to the sensor. The points shown for normal tactile sensing (Figure 5 and Figure 6) are the average of three measurement values against each applied normal pressure. Decreases in the resistance for all types of MWCNT/PDMS tactile sensors were observed as the applied pressure increased. Figure 5 illustrates the variation in R/R_o_ against the applied normal pressure for the ion beam treated MWCNT/PDMS films having surface with planar morphology, and vertical micropillars (5, 10, 20 μm high). The experimental results revealed that the tactile sensor with vertical micropillars of 20 μm is the most sensitive.

The sensitivities (S=(ΔR/Ro)/ΔP) of the tactile sensor with vertical micropillars of 10 μm and 20 μm were 2.06 kPa^−1^ and 21.7 kPa^−1^, respectively, while that of the tactile sensor with planar surface was 1.00 kPa^−1^ in the lower pressure regime (<0.3 kPa). The increased sensitivity, greater R/R_o_ change upon same amount of applied normal pressure, for the tactile sensor with 20 μm high vertical micropillars can be attributed by the additional deformation available because of the large pillar height. Thus, increasing the height of micropillars can be used as a way to enhance the sensitivity of tactile sensor skins. However, untreated 20 μm high vertical micropillars were liable to collapse during the repeated performance tests. Therefore, enhancement of stiffness of the micropillars must be used to prevent the collapse. The exposure of the MWCNT/PDMS film to Ar^+^ ion beam irradiation leads to the generation of a thin silica-like stiff layer of nanometer scale dimensions [36]. This thin stiff layer on the exposed surface provides enough stiffness for 20 μm vertical micropillars to withstand the repeated normal pressure providing the sensor durability. The lowered level of carbon concentration inside the surface layer after ion beam treatment can explain the increase of the initial resistance R_o_ that eventually results in a higher R/R_o_ change (higher sensitivity) under the same external pressure.

#### 4.1.2. Effect of Curvature of Micropillar

The 20 μm high micropillars were curved to various degrees after Ar^+^ ion beam irradiation. To control the curvature of the micropillars, the incident angle of the ion beam, exposure time, flow rate of argon gas and electrical current were optimized. FESEM images (Figure 4) shows the portion of the micropillars where wrinkles appear due to bombardment with the low energy Ar^+^ ion beam. In case of the micropillars exposed to the Ar^+^ ion beam at an incident angle of 0°, the Ar^+^ ion beam is parallel to the vertical micropillars (perpendicular to the substrate) and the tip of the micropillars is exposed to the ion beam while the sidewalls of the micropillars are not, which leads to the hardening of the micropillars only at the tip and the floor of MWCNT/PDMS film. In case of the micropillars exposed to the Ar^+^ ion beam at an incident angle of 30°, only the upper part of the micropillars’ sidewall is exposed to the ion beam while the bottom portion along the height of the micropillar is not, which results in a small curvature for the 20 μm high micropillars. The sensitivities of the tactile sensors having 20 μm high micropillars curved at various degrees were investigated with applied normal pressure. Figure 6 illustrates the comparison of R/R_o_ against the applied normal pressure for the Ar^+^ ion beam treated tactile sensor at incident angles of 0°, 30° and 60°. The highest sensitivity was observed with the tactile sensor irradiated at 0° incident angle, which has straight micropillars only hardened on the tip area. The increased sensitivity in case of the MWCNT/PDMS tactile sensors having straight micropillars as compared to those having the curved micropillars can be explained by the additional deformation possible under the same applied normal pressure since the sidewalls of the straight micropillars are not hardened due to ion beam treatment as well as the relatively large gap between film and electrode provided due to the high height of the micropillars. The measured sensitivities for the MWCNT/PDMS tactile sensor skins irradiated at 0°, 30° and 60° were 21.7 kPa^−1^, 14.0 kPa^−1^ and 7.63 kPa^−1^ in the lower pressure regime (<0.3 kPa), respectively.

### 4.2. Shear Tactile Sensing Characteristics

**Figure 7:**
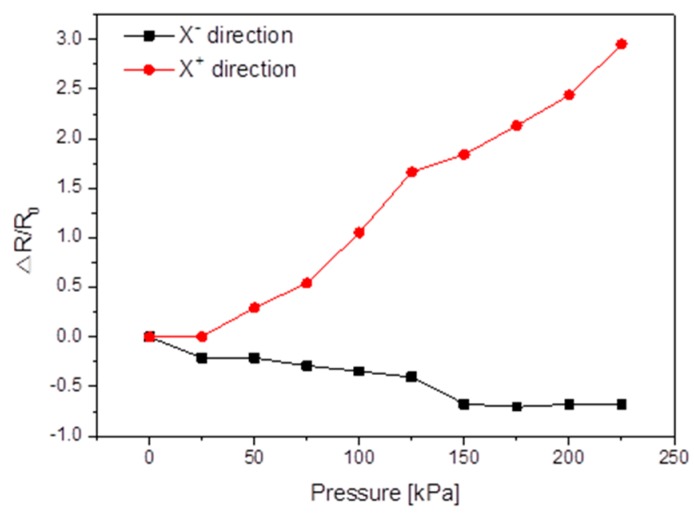
Shear pressure response of the MWCNT/PDMS tactile sensor having interlocking curved micropillars after Ar^+^ ion beam treatment at the incident angle of 60°.

For testing the shear pressure sensing capability, two MWCNT/PDMS films were arranged to have interlocked micropillars. The MWCNT/PDMS tactile sensor with interlocked micropillars was placed between two conductive electrodes in the presence of a normal preload to avoid any slippage during the shear pressure sensing tests. The shear pressures were applied by continuously moving the XY stage in X+ and X− direction at the speed of 10 μm/min and the resistance changes in the tactile sensor were monitored by measuring the resistance between the two electrodes. For shear tactile sensing, each point in Figure 7 is the average of 20 measurements against applied shear pressure. The contact area change of the interlocked micropillars upon applied shear pressure resulted in the resistance change of the proposed MWCNT/PDMS tactile sensor with interlocking curved micropillars. Since the micropillars irradiated at 60° have the maximum curvature, they were deformed easily under a small shear pressure and were three times more sensitive as compared to other types of curved MWCNT/PDMS tactile sensors. These phenomena can be understood because the sidewalls of the micropillars facing the ion gun are completely exposed to the 60° ion beam and the mismatch between the different stiffnesses of the thin layer on the MWCNT/PDMS film surface produces micropillars bending towards the direction of the Ar^+^ ion beam, so when the shear pressure was applied in the reverse direction of curving of top layer micropillars (X+), the contact area between the interlocked micropillars decreased leading to the increase in the resistance of the tactile sensor, while when the shear pressure was applied in the curving direction of the top layer micropillars (X−), the contact area of the interlocked micropillars increased resulting in a decrease in resistance of the proposed tactile sensor (Figure 7). The developed sensor is able to detect not only magnitude but also differentiate the direction when the applied shear pressure is applied along or opposite to the direction of curvature of the micropillars over the MWCNT/PDMS substrate. The sensing capabilities of the proposed tactile sensor can be further extended for the realization of tactile sensing mechanisms for prosthetic arms or legs, touch panels, wearable artificial skins, *etc.*

## 5. Conclusions

Inspired by the sensing capabilities of human skin, we have designed a MWCNT/PDMS piezoresistive tactile sensor having micropillars on the surface capable of detecting normal and shear pressure. The micropillar structured MWCNT/PDMS tactile sensor showed that increasing the height of micropillars enhanced the sensitivity under applied gentle touching. A low energy Ar^+^ ion beam treatment was performed to overcome the collapse problems associated with high micropillars (20 μm) and successfully enhanced the sensitivity of MWCNT/PDMS tactile sensor showing a sensitivity of 21.7 kPa^−1^ under gentle touch. These results originate from the formation of a stiff and stable layer and the increased initial resistance of the exposed surface of MWCNT/PDMS after ion beam treatment. Ar^+^ ion beam irradiation could also vary the curvature of the micropillars according to the incident angle of the ion beam. The curvature of the micropillars acts as another control factor in tuning the sensitivity under shear pressure. The interlocking curved micropillar MWCNT/PDMS tactile sensor showed its capability of the directional discrimination depending on the fastening and untangling motion of the interlocking structures. Further research is required to incorporate more sensitivity under the applied shear pressure for the realization of ultra-sensitive shear tactile sensors that can demonstrate their capability even under normal preloads comparable to a gentle touch.
